# Bovine colostrum supplementation and upper respiratory symptoms during exercise training: a systematic review and meta-analysis of randomised controlled trials

**DOI:** 10.1186/s13102-016-0047-8

**Published:** 2016-07-26

**Authors:** Arwel W. Jones, Daniel S. March, Ffion Curtis, Christopher Bridle

**Affiliations:** 1 Lincoln Institute for Health, University of Lincoln, Brayford Pool, Lincoln, LN6 7TS UK; 2 Department of Infection, Immunity and Inflammation, University of Leicester, Leicester, UK

**Keywords:** Athletes, Bovine colostrum, Exercise training, First milk, Respiratory illness, URTI

## Abstract

**Background:**

Bovine colostrum is proposed as a nutritional countermeasure to the risk of upper respiratory symptoms (URS) during exercise training. The aim of this systematic review and meta-analysis was to estimate the size of the effect of bovine colostrum supplementation on URS.

**Methods:**

Databases (CDSR, CENTRAL, Cinahl, ClinicalTrials.gov, Current Controlled Trials, DARE, EMBASE, Medline, PROSPERO and Web of Science) of published, unpublished and ongoing studies were searched for randomised controlled trials of healthy adults (≥18 years), evaluating the effect of oral bovine colostrum supplementation compared to a concurrent control group on URS.

**Results:**

Five trials (152 participants) met the inclusion criteria, all of which involved individuals involved in regular exercise training. Over an 8–12 week follow-up period, bovine colostrum supplementation when compared to placebo significantly reduced the incidence rate of URS days (rate ratio 0.56, 95 % confidence intervals 0.43 to 0.72, *P* value < 0.001) and URS episodes (0.62, 0.40 to 0.99, *P* value = 0.04) by 44 and 38 % respectively. There were limited data and considerable variation in results of included studies for duration of URS episodes hence a meta-analysis of this outcome was deemed inappropriate. The risk of bias assessment in this review was hindered by poor reporting practices of included studies. Due to incomplete reporting of study methods, four of the five studies were judged to have a moderate or high risk of overall bias. Our findings must be interpreted in relation to quantity and quality of the available evidence.

**Conclusions:**

The present systematic review and meta-analysis provides evidence that bovine colostrum supplementation may be effective in preventing the incidence of URS days and episodes in adults engaged in exercise training. The fact that the majority of included studies did not report significant effects on URS outcomes mitigates concerns about publication bias. The point estimates of the random-effects meta-analyses are greater than the smallest clinically important difference, but the low precision of the individual study estimates means the evidence presented in this review needs to be followed up with an appropriately designed and adequately powered, randomised control trial.

**Trial registration:**

Protocol was registered (CRD42015014925) on the International Prospective Register of Systematic Reviews (http://www.crd.york.ac.uk/PROSPERO/).

**Electronic supplementary material:**

The online version of this article (doi:10.1186/s13102-016-0047-8) contains supplementary material, which is available to authorized users.

## Background

Upper respiratory tract infections represent a leading cause for consultations in primary health care [1]. Lifestyle and behavioural factors are key determinants of infection risk [2]. Evidence from cross-sectional and longitudinal studies suggests that athletes are susceptible to symptoms of upper respiratory tract infections during periods of strenuous exercise training and immediately after competition [3–7]. Given the lack of feasibility in identifying pathogenic causes of infections in most research settings [1], the majority of findings are limited to self-reporting by athletes whereby upper respiratory symptoms (URS) has become the accepted reporting standard in exercising populations [8]. Regardless of whether URS are due to infectious causes or other inflammatory stimuli (allergies, airway irritation) mimicking infection, the potential for a negative impact (e.g. impaired performance) on the individual athlete may be the same [8, 9]. Despite being an active and rapidly growing area of research, there remains a lack of effective countermeasures to risk of URS during exercise training [10]. There is a clear need for evidence-based interventions that can reduce the burden of URS in at risk populations such as endurance athletes.

Bovine colostrum (BC) is the initial milk produced by a cow in the first few days following parturition. In contrast to mature milk, BC is rich in bioactive components including growth factors (e.g. epidermal growth factor), immunological mediators (e.g. immunoglobulins) and antimicrobial peptides (e.g. lactoferrin) that are homologous to human colostrum. The greater concentrations of these bioactive constituents in BC have led to proposed benefits towards human immune health [11]. Unlike the passive transfer of immunity that is important for the development of neonatal calves, it is proposed that the small bioactive constituents of BC, which survive digestion, or their metabolites that appear after consumption, have direct effects on human immunity [12]. A recent systematic review exploring the potential applications of BC (including risk of URS) concluded that BC supplementation may enhance or protect host defence under certain detrimental situations (e.g. exercise-induced immune dysfunction) but the exact clinical benefits are yet to be established [13]. However, findings from this review should be interpreted with caution as data of included studies were not statistically combined to quantify effect size, we are also aware of at least one other study [14] on the risk of URS that was not included.

The current evidence of the scientific validity of BC is limited, and the level of evidence used in support of its claims falls below that acceptable in the medical community. While the evidence indicates that BC may have a role in reducing the risk of URS, there is currently no systematic review and meta-analysis that has synthesised the effect of BC interventions on URS. The aim of this study was to systematically review randomised controlled trials in order to estimate the size of the effect of BC supplementation on URS. Specifically, we examined whether BC supplementation reduces the incidence rate of URS days or episodes and duration of URS episodes during exercise training.

## Methods

Methods of analysis and inclusion criteria were specified in advance and documented in a protocol that was registered (CRD42015014925) on the International Prospective Register of Systematic Reviews (http://www.crd.york.ac.uk/PROSPERO/).

### Eligibility criteria

This review considered studies for inclusion if they were randomised controlled trials, evaluating the effect of oral supplementation of non-hyperimmune BC on URS outcomes among healthy adults (≥18 years).

### Study identification

To identify any existing relevant systematic reviews, published and ongoing trials the following electronic databases were searched from inception to July 2015: Cinahl, ClinicalTrials.gov, Cochrane Central Register of Controlled Trials; CENTRAL, Cochrane Database of Systematic Reviews; CDSR, Current Controlled Trials, DARE, EMBASE, Medline, PROSPERO and Web of Science. We also searched the British library (ETHOS) and other library services within our Institution for obtaining non-published data. No restrictions on language were imposed. Key terms used to search trials registers and databases were terms for BC interventions (e.g. bovine colostrum, see Additional file 1). Database searching was supplemented with internet searching (e.g. Google Scholar), forward and backward citation tracking from systematic reviews and included studies, and contact with study authors, experts and research groups. Search results were downloaded to Endnote. Duplicate citations were removed and titles and abstracts screened independently by two reviewers against the inclusion criteria. Where studies could not be excluded based on title or abstract, two reviewers assessed full text papers for relevance independently. Any discrepancies were resolved through discussion, or where required a third reviewer.

### Data abstraction

We developed, tested and refined a structured data extraction template. For each study, one reviewer extracted data, which was cross-checked for accuracy by a second reviewer. Information was extracted from each included trial on: characteristics of participants (age, gender, inclusion criteria, exclusion criteria); type of intervention (non-hyperimmune BC vs placebo, dose, duration, frequency/timing, diluter); type of outcome measure (self-reported total number of days with URS, number of URS episodes, duration of episodes).

The Cochrane risk of bias assessment tool was used to assess studies for accuracy of key quality issues such as method of randomisation, blinding and follow up [15]. Each domain was classified as adequate, unclear or inadequate and the overall risk of bias for each study was interpreted as follows: low risk of bias, (all criteria graded adequate); moderate risk of bias (one criterion graded inadequate or two graded unclear) and high risk of bias (more than one criterion inadequate, more than two graded unclear). As only five studies were included, all four reviewers examined and agreed on the risk of bias for each domain for each included study.

### Data analysis

All analyses were conducted using Review Manager version 5.3. The primary outcome measure was the rate ratio of URS days. The secondary summary measures were the rate ratio of episodes of URS and the mean duration of an episode of URS. We contacted study authors to obtain missing numerical outcome data and in cases where studies only reported URS as one of the above outcomes, we verified that no additional data were available. Rate ratio of URS days and episodes of URS were calculated using the incidence rate in BC groups divided by incidence rate in placebo groups. The incidence rate for each group was based on the total number of URS days or (study defined) URS episodes divided by the total number of days of observation. The (natural) logarithms of the rate ratios and the standard error of the rate ratio were combined statistically using the generic inverse-variance random-effects model according to [15]. Duration of URS episodes was analysed as a continuous outcome and expressed as the between-groups difference in mean duration of episodes. Random-effect models were used in all meta-analyses, as they are more conservative than the fixed effects models since, by incorporating within- and between-study variance, the confidence intervals for the summary effect are wider. This is based on the underlying assumption that the individual study estimates are not identical but follow a normal distribution. Statistical heterogeneity in any random-effect meta-analyses was assessed by the I^2^ test [15]. The I^2^ statistic indicated the proportion of variation among effect estimates above that expected by chance. In cases where heterogeneity was considered to be important (I^2^ > 40 %) [15], sources of clinical and methodological diversity were explored. We attempted to explore the effect of BC on the primary outcome (rate ratio of days of URS) in pre-specified subgroups of study stratified by dose (high versus low) or duration of intervention. The robustness of results was assessed in separate sensitivity analyses that excluded trials with high risk of bias.

## Results

**Fig. 1:**
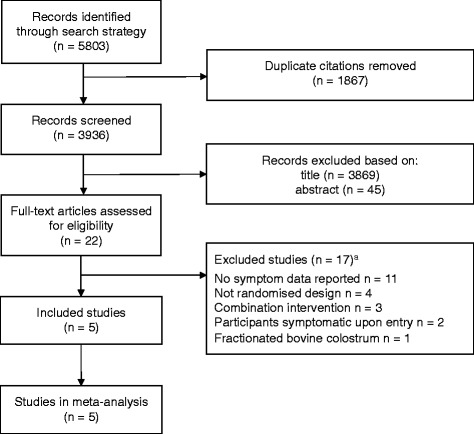
Flow diagram of study selection. ^a^Some studies excluded for multiple reason.

After removal of duplicates, the search strategy identified 3936 distinct citations, of which 3914 were excluded during the initial screening phase (Fig. 1). For the remaining 22 citations, full-text papers were ordered, obtained and independently assessed against the eligibility criteria, with two discrepancies resolved by discussion. Studies were included if the allocation to BC and control groups were randomised or based on a quasi-random method. Five studies (23 %) met the inclusion criteria [14, 16–19]. The main reasons for exclusion of full-text papers were studies not reporting URS outcomes (*n* = 11), non-randomised study designs (*n* = 4) and BC combined with other interventions (*n* = 3).

### Characteristics of included studies

The five included studies were published between 2006 and 2014 (Table 1). Of the five studies, two were conducted in Australia [18, 19], two in New Zealand [16, 17] and one in Wales [14]. One of these trials was explicitly identified in the study report as being a pilot study [19]. The five trials randomised 152 participants (80 % males), with sample size ranging from 10 to 53.

**Table 1:** Characteristics of included studies

Study (country)	Sample size	Age, years	Proportion of males	Inclusion criteria	Exclusion criteria	Intervention, dose, frequency/timing, duration, diluter	Placebo
Crooks et al. 2006 [16] (New Zealand)	BC: 18 PLA: 17	Median (range) BC: males, 46 (35–57), females, 43 (30–53)	0.48	Pack runs ≥ 1 week, marathon training over last 5 years, age < 60 year	Lactose intolerant, allergy to cows milk, whey-protein supplements, treatment for any diagnosed condition	Oral BC (Immulac, NZMP Ltd, Auckland, New Zealand), 26 g of powdered sachets/day corresponding to 10 g of BC, daily, 12 weeks, chocolate powder with 125 ml water	Skim milk matched for equivalent digestible protein content. Flavours and colours added to match BC
		PLA: males, 48 (36–56) females, 51 (41–58)					
Crooks et al. 2010 [17] (New Zealand)	BC: 12 PLA: 13	Mean ± standard error BC: males, 17 ± 1 females, 20 ± 1	0.57	Participating in training program prior to The Auckland Swimming Championships	Lactose intolerant, allergy to cows milk, whey-protein, immunological-modulating supplements, treatment for any diagnosed condition	Oral BC (Immulac, NZMP Ltd, Auckland, New Zealand), 52 g of powdered sachets/day corresponding to 20 g of BC, 10 g morning & evening, 10 weeks, 125 ml water	Skim milk powder, matched for equivalent protein, fat and carbohydrate content
		PLA: males, 19 ± 1, females, 18 ± 1					
Jones et al. 2014 [14] (Wales)	BC: 25 PLA: 28	Mean ± standard deviation BC: 31 ± 14 PLA: 32 ± 13	1.00	≥3 h moderate-vigorous endurance exercise/week	Smoker, medication or other supplements, infectious illness in 4 weeks prior to study	Oral BC (Neovite, UK, London), 20 g/day, 10 g with morning & evening meal, 12 weeks	Isoenergetic/isomacronutrient PLA as described elsewhere
Shing et al. 2007 [18] (Australia)	BC: 14 PLA: 15	Mean ± standard error BC: 29 ± 1 PLA: 27 ± 2	1.00	Cyclists racing competitively ≥ 2 months, consistent training volumes ≥ 2 months	Dietary supplements 1 month prior to study	Oral BC (Numico Research Australia, South Australia, Australia), 10 g/day, morning, 8 week 1 day, 50 ml water + 100 ml milk	Whey protein (Alacen 80; Fonterra Co-op group Limited, Auckland, New Zealand)
Shing et al. 2013 [19] (Australia)	BC: 4 PLA: 6	Mean ± standard error BC: 22 ± 3 PLA: 23 ± 2	1.00	Cyclists racing competitively ≥3 months. Consistent training volumes ≥ 2 months	None reported	Oral BC (Numico Research Australia, South Australia, Australia), 10 g/day, morning, 8 weeks and 5 days, 50 ml water + 100 ml milk	Whey protein concentrate (Alacen 80; Fonterra Co-op group Limited, Auckland, New Zealand)

*BC* bovine colostrum, *PLA* placebo

Participants within all included trials were regularly involved in endurance exercise, defined by training volume and/or participation in a training program for a competitive event. Participants were highly trained cyclists [18, 19], recreational distance runners [16], elite swimmers [17] or those recreational active [14] from a range of endurance-based training (running, cycling, swimming, triathlon, team games). In-training eligibility was determined by self-report of exercise training in three [14, 16, 17] of the studies, and membership of a team that was ‘in-training’ in two [18, 19] of the studies.

Participants during all trials self-reported physical activity. Four of the five studies used daily training diaries/logs [16–19], and one study [14] required participants to complete the international physical activity questionnaire on a weekly basis. Aside from the supplementation, participants in all of the trials continued with their usual diets during trial periods.

Common participant exclusion criteria in the individual trials were the use of supplements [14, 16–18], intolerance to lactose [16, 17] or use of medication [14, 16, 17]. One study [14] also stipulated that participants should be non-smokers and free from infectious illness for 4 weeks prior to the study.

All trials compared BC to placebo. Supplementation period in these trials was 8 weeks [18, 19], 10 weeks [16], or 12 weeks [14, 17]. All studies included provision of BC in powdered form where daily dosage of the BC intervention was either 10 g [16, 18, 19] or 20 g [14, 17]. In two of the trials [18, 19], the placebo comprised of whey protein, two trials [16, 20] used skimmed milk, which was consumed at the identical dose, frequency, and duration as the BC supplement. One trial [14] reported use of a placebo in line with a previously published study [20]; an isoenergetic/isomacronutrient mixture of milk protein concentrate and skimmed milk powder. BC and placebo powdered supplements were isocaloric in three studies [14, 17, 19] and matched for digestible protein content in one trial [16]. Although each study reported to be a double-blind trial, none assessed the extent to which participants may have deduced their group allocation / treatment regimen.

Outcomes relevant to incidence or duration of URS were not specified as primary outcomes in any of the five trials, although three [14, 16, 17] did include them within their “main outcomes”. Pre-specified checklists, self-reported by participants, were used to record URS in all of the trials but a physician did not verify these medically. Studies used measurement instruments that were either previously published [14, 16, 17] or validated (Wisconsin Upper Respiratory Symptom Survey [18, 19]. URS days were included in the analysis within each of the included studies when two or more consecutive days were reported. Although none of the included studies undertook pathogenic aetiology of symptoms, three studies had additional criteria for URS to be classified as an episode. Two studies [18, 19] required the presence of two or more URS to be recorded (e.g., runny or stuffy nose, sore or scratchy throat, sneezing, headache, fever, cough) while the other [14] classified an episode on a pre-defined symptom score which ultimately required at least three symptoms (sore throat, catarrh in the throat, runny nose, cough, repetitive sneezing, fever, persistent muscle soreness, joint aches and pains, weakness or headache) of a moderate severity to last for a minimum of 2 days, or two moderate symptoms to last for 3 days. Included studies required participants to be free of symptoms for a period of 3 days [16] or 7 days [14, 18, 19] before an URS episode was considered separate to a previous episode. Number of URS days or episodes and duration of episodes were treated as continuous outcomes in all of the studies and interpreted as a difference in means. Comparisons in the proportions of BC and placebo groups to report incidence of URS was also presented by two studies [14, 17].

The included studies varied for risk of bias (Table 2). Three of the five studies were considered to have a high overall risk of bias [14, 16, 17], one was moderate [19] and one low risk of bias [18]. The risk of bias assessment was hindered by poor reporting. No individual domain across all studies was graded inadequate for risk of bias. It was an accumulation of several domains classified as unclear (due to insufficient reporting) that resulted in an overall moderate or high risk of bias for included studies.

**Table 2:** Risk of bias within trials

Study or subgroup	Random sequence generation	Allocation concealment	Blinding: participants and personnel	Incomplete outcome data	Overall risk
Crooks 2006 [16]	unclear	unclear	unclear	adequate	high
Crooks 2010 [17]	unclear	unclear	unclear	adequate	high
Jones 2014 [14]	unclear	unclear	unclear	adequate	high
Shing 2007 [18]	adequate	adequate	adequate	adequate	low
Shing 2013 [19]	adequate	adequate	adequate	unclear	moderate

### Effect of bovine colostrum on upper respiratory symptoms (URS)

#### Rate ratio of URS days

**Fig. 2:**
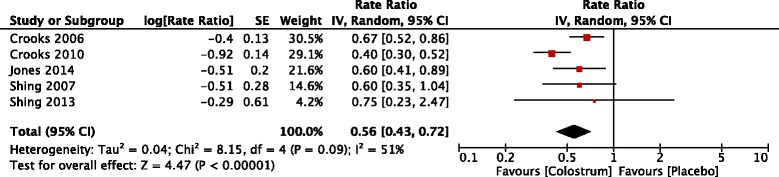
Trial-level data, effect estimates and forest plot of comparison for the rate ratio of days with upper respiratory symptoms

Five trials reported the number of URS days [14, 16–19]. There were 73 participants in the BC group and 79 in the placebo group. The point estimate of effect for each of the included trials indicated a lower incidence rate of URS days with BC. Pooled analyses from the five trials demonstrated a significant effect of BC supplementation on the rate of URS days (rate ratio 0.56, 95 % confidence intervals 0.43 to 0.72, *P* value < 0.001) (Fig. 2). Moderate level of statistical heterogeneity was detected among trial level effects when all five trials were included (Chi^2^ = 8.15; df = 4, P value = 0.09; I^2^ = 51 %). Compared to other included studies, one study [18] was limited to swimmers only. Removal of Crooks [18] reduced the I^2^ to zero but the effect of BC on URS days remained significant (0.64, 0.53 to 0.78, *P* value < 0.001).

#### Rate ratio of episodes of URS

**Fig. 3:**
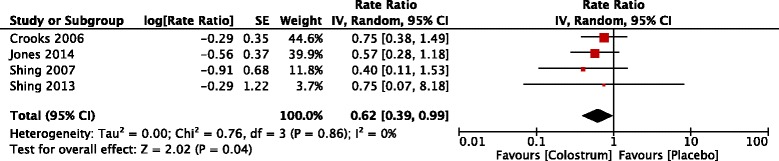
Trial-level data, effect estimates and forest plot of comparison for the rate ratio of episodes of upper respiratory symptoms

Four trials reported the number of URS episodes [14, 16, 18, 19]. There were 61 participants in the BC group and 66 in the placebo group. The point estimate of effect for each of the included trials indicated a lower incidence rate of URS days with BC. Pooled analyses showed that across these trials, the episode rates of URS were significantly lower with BC (rate ratio 0.62, 95 % confidence intervals 0.40 to 0.99, *P* value = 0.04) (Fig. 3). Statistical heterogeneity was not apparent in this outcome (Chi^2^ = 0.76; df = 3, *P* value = 0.86; I^2^ = 0 %).

### Duration of URS episodes

Three trials reported the duration of URS episodes [14, 18, 19]. There were 43 participants in the BC group and 49 in the placebo group. One trial [19] had insufficient data to be used in pooled analysis having observed a low number of URS episodes (one event, duration of 5 days) during BC supplementation (mean ± standard deviation of placebo group: 4 ± 1.4 days). The other two included trials reported shorter [14] (Mean difference −2.0, 95 confidence intervals −4.7 to 0.7) or longer [18] (2.1, 0.2 to 4.0) duration of URS episodes with BC supplementation. Given the limited data and considerable variation in results, particularly the inconsistency in the direction of effect, a meta-analysis of this outcome was deemed inappropriate.

### Subgroup and sensitivity analyses

There were too few studies for meaningful synthesis of subgroups and sensitivity analyses of our primary outcome (URS days). However, if further data become available, these should be included in a future meta-analysis. In particular, the trend for larger effects in trials [14, 16] using a daily BC dose of 20 g (rate ratio 0.48, 95 % confidence intervals 0.32 to 0.71) compared to trials using 10 g [17–19] (0.66, 0.53 to 0.83, *P* value < 0.001) warrants further attention.

## Discussion

### Summary of main findings

The review identified five randomised controlled trials [14, 16–19] evaluating the short-term effects of BC on URS during exercise training. Synthesised data from five [14, 16–19] trials showed a statistically significant effect in which, compared to placebo, BC reduced the rate of URS days by 44 %. Similarly, synthesised data from four trials [14, 16, 18, 19] indicated statistically significant effects favouring BC with a 38 % reduction in episode rates of URS.

### Strengths and weaknesses of the study

The study adhered to the pre-specified protocol using appropriate methods to select, evaluate and synthesise all the relevant evidence. A comprehensive search for published and unpublished studies, which included multiple electronic databases, scanning of bibliographies and contact with authors yielded five published studies. To date there are only a limited number of randomised controlled trials in this area with small sample sizes. A strength of this review is that it presents the first meta-analyses synthesising the effects of BC supplementation on URS. The point estimates for the effect of BC on the rate of URS days and episodes demonstrate a small [21], but clinically important reduction. The estimate of the effect of BC on URS did indicate the presence of a moderate statistical heterogeneity. Whilst we identified a potential source of heterogeneity, the inclusion or exclusion of this individual study estimate would not modify the conclusions drawn in this review. The small sample sizes and hence the low number of events (symptoms, episodes) led to wide confidence intervals for the point estimates. For the reduction in URS days, the lower confidence limit is still greater than the smallest clinically important difference (ratio 0.90; 10 % change in incidence) [22]. As the entire range of the confidence interval for the effect of BC on URS episodes does not exceed the threshold of clinical usefulness, we cannot exclude the possibility that the reduction in episodes is of a magnitude not considered clinically worthwhile.

The lack of identification and hence inclusion of unpublished evidence is often considered a limitation in systematic reviews. Effects estimated from published studies may be inflated due to bias towards the non-publication of studies with non-significant effects. We did not have a sufficient number of individual study estimates to use a funnel plot to detect such bias in this review. However, the fact that the majority of included studies did not report significant effects on URS outcomes mitigates concerns about publication bias. The decision to publish appears to be independent of an observed effect. There was potential for bias in all of the trials included in this meta-analysis, however no individual risk of bias domain was classified as high risk. There were many items where the risk of bias was unclear, primarily due to incomplete reporting by authors. Consequently, there is a degree of uncertainty in relation to bias for the included evidence.

The incidence of URS in the included studies in this review was on a self-reported basis only. Although incidence during exercise training and/or around periods of competition has long been considered to be of an infectious origin, both pathogen identification and physician confirmation of URS are uncommon in research settings [8]. Due to the large number of potential causative pathogens [1], and hence the cost associated with such diagnostic procedures, pathogen identification is not always feasible [8]. Physician diagnosis, once considered the ‘gold standard’, has also come under scrutiny due to discrepancies with laboratory evaluation of bodily fluids from symptomatic individuals [3]. It has been argued, that self-diagnosis (at least of infections) and not physician diagnosis is the most reasonable approach for research studies given the familiarity of the general population with such symptoms [23]. However, the limited range of physiological responses within the upper respiratory tract means that there are similarities in the early signs and symptoms (sore throat, sneezing, runny nose, and nasal congestion) of airway irritation, infection and allergy [23, 24]. The included studies had made further attempts to at least aid the interpretation of the underlying cause of URS (defining the minimum number of days/symptoms before consideration). However, as there was no pathological investigation, we cannot be certain that all self-reported URS were of an infectious cause. Therefore, we have maintained the use of URS (as opposed to upper respiratory tract infection, URTI) in this review, in line with the recommendations placed within a position statement led by experts of the International Society of Exercise Immunology [8].

All of the included studies were randomised, placebo-controlled trials. Two [16, 17] of the included trials reported that they used skimmed milk for a placebo, two [18, 19] used whey protein and one [14] used an isoenergetic and isomacronutrient mixture of skimmed milk and milk protein concentrate. Each of the included studies provided their respective BC and placebo groups with similar amounts of protein. The only difference between the placebo and test drug should be the absence of a putative active or characteristic feature [25]. Any negative, positive, or same direction placebo effects could result in the appearance of misleading negative, positive, or non-significant effects of the experimental substance. An effect of BC has been demonstrated despite use of placebo substances (whey peptides) within included studies [18, 19] that also possess immune-modulating properties [26] and hence potential to benefit URS outcomes.

### Comparison with other studies

There is currently no meta-analysis that has assessed the effect of BC on URS. We are not aware of another review evaluating the effect of BC on URS in healthy adults, although one review does discuss the clinical applications of BC therapy [13]. The aim of the previous review [13] was to evaluate the evidence in relation to the effect of BC on any quantifiable change in a health or performance related clinical or paraclinical parameter. Unlike the current review, Rathe et al. [13] did not include the use of a control group as a criterion for inclusion of individual studies, nor did they statistically combine the findings of these studies into a single numerical estimate of effect. Also, the three-item jaded scale was used to evaluate the methodological quality of the included studies, which has often been criticised for its over-simplification of the process, and a greater focus on reporting quality rather than methodological quality. The present study reduces uncertainty concerning the clinical effect of BC.

### Future research

The low precision of the individual study estimates (as a result of small sample sizes and hence low number of events) within the present meta-analyses widens the confidence intervals for the point estimate of the effect of BC. The evidence presented in this review needs to be followed up with an appropriately designed and adequately powered, randomised control trial. None of the included studies reported an a priori sample size calculation to determine the effect of BC on URS. Majority of the studies were limited by expressing URS incidence as a continuous outcome measure and interpreting the effects of BC on mean differences. The incidence of URS days and episodes are count data whereby authors should express the outcomes as rates or ratios and analyse using a Poisson regression model with an overdispersion correction. Future studies should include an a priori sample size calculation, powered and justified on the smallest clinically important effect which has been proposed to be a 10–20 % change in the incidence of illness (ratio of 0.9/0.83) [22].

It has long been considered that transient perturbations in host immunity following strenuous training and/or competition predispose athletes to an “open window” of increased susceptibility to infectious (pathogenic) causes of URS [27]. More recently, non-infectious (e.g. airway irritation, allergies, asthma) hypotheses have been proposed to explain a proportion of the incidence of self-reported URS following exercise [8]. Given the uncertainty surrounding the underlying causes of URS with exercise [8], there may be a number of potential mechanisms responsible for the effects of BC on URS during exercise training. An in-depth discussion of underlying mechanisms of the effects of BC is beyond the scope of this review. The current available evidence does at least suggest that BC counters perturbations in intestinal barrier integrity, immune cell functions and salivary antimicrobial peptides (AMPs) following strenuous exercise [12]. Future studies should consider biologically sensitive, clinically relevant in vivo markers of immunity to support interpretation [28], although immune measures should always be viewed as secondary parameters that complement primary (clinical symptom) endpoints and not vice versa. Although it is the presence of symptoms that are perceived as a nuisance at the individual level, not the infection per se [1], it would be prudent for future research, where possible, to conduct more exhaustive clinical screening of participants upon study entry and perform pathological testing to identify infectious from non-infectious causes of URS. We did not synthesise the effect of BC on duration of URS episodes, further trials are needed to measure this outcome and the severity of episodes, which was not considered in this review. These additions would not only help the interpretation of changes in URS with BC during exercise training, but also inform the medical and scientific community of other target populations (immunocompromised, inflammatory, allergic) that may benefit from BC supplementation.

There were a limited number of studies to undertake a pre-planned analysis of trials stratified by dosage and duration of BC intervention, but tentative evidence provided in this review suggests that there may be a dose-response effect of BC. The reduction in the incidence rate of URS days with BC on average was larger (by 18 %) in studies using 20 g compared to 10 g. It is worthy to note that this finding is purely observational by nature whereby a head to head randomised comparison of different doses is required. Research to confirm the minimum and optimum dose for the effect of BC on incidence and duration of URS should be considered. It has previously been suggested that some of the immune-modulatory effects of BC are due to bioactive components that become biologically active upon digestion of BC [12]. Dose alone is not the only factor that could lead to variation in the magnitude of exposure to such bioactive (health-promoting) components. Composition of these bioactive components is superior during the first milkings and subsequently decreases during the first 3 days of lactation to those of mature milk [29]. In order to further clarify the effects of BC between studies, investigations should specify collection and processing methods of BC (e.g. timing of collection period) and provide a composition profile of the product (i.e. provide evidence of product quality such as components found in early milkings).

Our findings must be interpreted in relation to quantity and quality of available evidence. The risk of bias assessment in this review was hindered by poor reporting practices of included studies. Although the quantity and quality of the evidence was less than ideal, we do not consider these limitations to be sufficient to dismiss the findings of this review. However, future trials are urged to improve on the current reporting of parallel-group randomised controlled trials on BC by adhering to Consolidated Standards of Reporting Trials (CONSORT) guidelines that facilitate critical appraisal and interpretation.

In general BC supplementation is considered to be safe and well tolerated in humans [30]. One study included in this review reported side effects presenting as stomach problems, in females only, which were mild and generally disappeared with time [16]. Another study indicated no side effects with BC supplementation [17]. None of the other included studies reported side effects or adverse reactions following BC supplementation. However, it is unclear if this was due to lack of incidence or a lack of measurement within the studies. Future research is recommended to monitor and report findings on side effects to help establish the safety profile of BC and its applicability as an intervention in other (e.g. clinical) populations.

## Conclusion

This systematic review and meta-analysis shows that oral supplementation of BC reduces the incidence rates of URS days and episodes in adults involved in exercise training. This may have important implications for the field of sports medicine, which to date has limited evidence for supporting the use of nutritional countermeasures against URS under conditions of physiological stress. In the absence of side effects, future prospective studies are recommended in other at risk groups (e.g. elderly and immunocompromised populations) to further establish the public health impact of BC.

## Abbreviations

BC, bovine colostrum; CONSORT, consolidated standards of reporting trials; URS, upper respiratory symptoms

## Additional file

Additional file 1: Sample search strategy (PDF 31 kb)

## References

[1] Eccles R (2005). Understanding the symptoms of the common cold and influenza. Lancet Infect Dis.

[2] Walsh NP, Gleeson M, Pyne DB (2011). Position statement. Part two: Maintaining immune health. Exerc Immunol Rev.

[3] Cox AJ, Gleeson M, Pyne DB (2008). Clinical and laboratory evaluation of upper respiratory symptoms in elite athletes. Clin J Sport Med.

[4] Hellard P, Avalos M, Guimaraes F (2015). Training-related risk of common illnesses in elite swimmers over a 4-yr period. Med Sci Sports Exerc.

[5] Nieman DC, Henson DA, Dumke CL (2006). Relationship between salivary IgA secretion and upper respiratory tract infection following a 160-km race. J Sports Med Phys Fitness.

[6] Heath GW, Ford ES, Craven TE (1991). Exercise and the incidence of upper respiratory tract infections. Med Sci Sports Exerc.

[7] Peters EM, Bateman ED (1983). Ultramarathon running and upper respiratory tract infections. An epidemiological survey. S Afr Med J.

[8] Walsh NP, Gleeson M, Shephard RJ (2011). Position statement. Part one: Immune function and exercise. Exerc Immunol Rev.

[9] Schwellnus MP, Lichab M, Derman EW (2010). Respiratory tract symptoms in endurance athletes - a review of causes and consequences. Curr Allergy Clin Immunol.

[10] Gunzer W, Konrad M, Pail E. Exercise-induced immunodepression in endurance athletes and nutritional intervention with carbohydrate, protein and fat-what is possible, what is not? Nutrients. 2012;4:1187–212. 10.3390/nu4091187 PMC3475230 23112908

[11] Shing CM, Hunter DC, Stevenson LM (2009). Bovine colostrum supplementation and exercise performance: potential mechanisms. Sports Med.

[12] Davison G (2012). Bovine colostrum and immune function after exercise. Med Sport Sci.

[13] Rathe M, Muller K, Sangild PT (2014). Clinical applications of bovine colostrum therapy: a systematic review. Nutr Rev.

[14] Jones AW, Cameron SJ, Thatcher R (2014). Effects of bovine colostrum supplementation on upper respiratory illness in active males. Brain Behav Immun.

[15] Higgins JPT, Green S (editors). Cochrane Handbook for Systematic Reviews of Interventions Version 5.1.0. The Cochrane Collaboration. 2011. http://www.handbook.cochrane.org. Accessed 19 Nov 2015.

[16] Crooks CV, Wall CR, Cross ML (2006). The effect of bovine colostrum supplementation on salivary IgA in distance runners. Int J Sport Nutr Exerc Metab.

[17] Crooks C, Cross ML, Wall C (2010). Effect of bovine colostrum supplementation on respiratory tract mucosal defenses in swimmers. Int J Sport Nutr Exerc Metab.

[18] Shing CM, Peake J, Suzuki K (2007). Effects of bovine colostrum supplementation on immune variables in highly trained cyclists. J Appl Physiol.

[19] Shing CM, Peake JM, Suzuki K (2013). A pilot study: bovine colostrum supplementation and hormonal and autonomic responses to competitive cycling. J Sports Med Phys Fitness.

[20] Davison G, Diment BC (2010). Bovine colostrum supplementation attenuates the decrease of salivary lysozyme and enhances the recovery of neutrophil function after prolonged exercise. Br J Nutr.

[21] Hopkins WG (2010). Linear Models and Effect Magnitudes for Research, Clinical and Practical Applications. Sportscience.

[22] Hopkins WG (2006). Estimating sample Size for magnitiude-based inferences. Sportscience.

[23] Eccles R (2013). Is the common cold a clinical entity or a cultural concept?. Rhinology.

[24] Spence L, Brown WJ, Pyne DB (2007). Incidence, etiology, and symptomatology of upper respiratory illness in elite athletes. Med Sci Sports Exerc.

[25] Golomb BA, Erickson LC, Koperski S (2010). What’s in placebos: who knows? Analysis of randomized, controlled trials. Ann Intern Med.

[26] Nelson AR, Jackson L, Clarke J (2013). Effect of post-exercise protein-leucine feeding on neutrophil function, immunomodulatory plasma metabolites and cortisol during a 6-day block of intense cycling. Eur J Appl Physiol.

[27] Nieman DC (2000). Is infection risk linked to exercise workload?. Med Sci Sports Exerc.

[28] Albers R, Bourdet-Sicard R, Braun D (2013). Monitoring immune modulation by nutrition in the general population: identifying and substantiating effects on human health. Br J Nutr.

[29] Blum JW, Hammon HM (2000). Bovine colostrum: more than just an immunoglobulin supplier. Schweiz Arch Tierheilkd.

[30] Struff WG, Sprotte G (2008). Bovine colostrum as a biologic in clinical medicine: a review--Part II: clinical studies. Int J Clin Pharmacol Ther.

