# Supplementary material for: Bovine colostrum supplementation and upper respiratory symptoms during exercise training: a systematic review and meta-analysis of randomised controlled trials
Source: BMC Sports Sci Med Rehabil. 2016 Jul 26;8:21. doi: 10.1186/s13102-016-0047-8 (PMC4960812; doi:10.1186/s13102-016-0047-8)
Supplement: Additional file 1: — Sample search strategy (PDF 31 kb) [file 13102_2016_47_MOESM1_ESM.pdf]

Additional file 1

Bovine colostrum supplementation and upper respiratory tract symptoms during exercise training: a systematic review and meta-analysis of randomised controlled trials

Sample search strategy

	**Search term**
**1**	bovine colostrum
**2**	bovine colostrums
**3**	beastings
**4**	1 OR 2 OR 3
